# Death of Barent P. Staats, M. D., of Albany

**Published:** 1871-07

**Authors:** 


### Death of Barent P. Staats, M. D., of Albany.

Many of our readers will be pained to hear of the death of Dr. Staats, of Albany, F. Y., who died recently, in the 75th year of his age. He was one of the oldest and most respectable members of the medical profession in that city, and for many years an active and influential member of the State Medical Society. He received the confidence and support of the public in marked degree, and was honored by his fellow citizens with important positions of trust. In all the varied positions of his long and useful life, (having practised his profession fifty-four years,) he was ever true to his profession, faithful to his friends and the general good. His life was one of great usefulnees, and his memory will long be cherished by all who knew him, with affectionate regard.

